# Update on TDM (Therapeutic Drug Monitoring) with Ustekinumab, Vedolizumab and Tofacitinib in Inflammatory Bowel Disease

**DOI:** 10.3390/jcm10061242

**Published:** 2021-03-17

**Authors:** Sophie Restellini, Waqqas Afif

**Affiliations:** 1 Division of Gastroenterology, McGill University Health Centre, Montreal, QC H3G 1A4, Canada; sophie.restellini-kherad@mail.mcgill.ca; 2 Division of Gastroenterology and Hepatology, Geneva’s University Hospitals and University of Geneva, 1205 Geneva, Switzerland

**Keywords:** therapeutic drug monitoring, biologic, ustekinumab, vedolizumab, tofacitinib

## Abstract

The goal of therapeutic drug monitoring (TDM) is to optimize anti-TNF (tumor necrosis factor) biologic treatment in patients with inflammatory bowel disease (IBD). Although commercial assays are readily available for both ustekinumab and vedolizumab, the use of TDM with these newer biologic medications is at its infancy. The clinical utility of TDM with non-anti-TNF mechanisms of action is not clear. This review summarizes the latest available data on the pharmacokinetics of newer biologic and oral small molecules and highlights the threshold concentrations that have been associated with improved outcomes in IBD patients.

## 1. Introduction

Biologic therapies have become the state of art in the management of inflammatory bowel disease (IBD). The tumor necrosis factor (TNF) antagonists were the only class of biologic agents approved for almost 15 years. Recently, a growing array of biologic agents and small-molecule treatments have joined the medical arsenal [1], targeting different pathways of inflammation in both Crohn’s disease (CD) and ulcerative colitis (UC). Among these, vedolizumab (VDZ), a monoclonal antibody that inhibits the α4β7 integrin of the gut mucosal addressin cell adhesion molecule 1 (MAdCAM-1) and ustekinumab (UST) which is directed against the p40 protein subunit used by both the IL-23 and IL-12 cytokine, have been approved for UC and CD [2,3,4,5,6]. In addition, a new strategy of blocking the Janus kinases (JAKs)/signal transducer and activator of transcription proteins (STATs) signaling pathway has been developed, which targets the large number of cytokines operated by JAK signaling activation. In this category, tofacitinib is already available for UC and two other molecules are under the last phases of investigation for CD (filgotinib and upadacitinib) [7].

Therapeutic drug monitoring (TDM) has received considerable interest in the past years, in order to personalize the care of patients with IBD [8,9]. This strategy is used to monitor blood drug concentrations and immunogenicity. Immunogenicity is an important concept with biologic medications and refers to the idea that the immune system recognizes parts of the drugs as non-self and develops antidrug antibodies (ADA). These ADA inactivate the therapeutic effects of the treatment and may increase drug clearance, leading to lower drug concentrations with suboptimal response or loss of response (LOR).

TDM offers a window into individual patient pharmacokinetics and acts as a guide to choose the best therapeutic option in the event that a patient loses response to treatment (reactive strategy) or to avoid LOR (proactive strategy). The value of TDM in clinical practice is clear for anti-TNF medications, as multiple pivotal clinical trials have demonstrated that infliximab, adalimumab, golimumab and certolizumab pegol concentrations are higher in patients achieving clinical and endoscopic remission, in comparison to nonresponders. The target cutoffs varies between studies, different endpoints/drugs and timing of the assessment (induction or maintenance period) [10]. Various recommendations call for use of TDM to lead treatment choices regarding both proactive and reactive treatment management [10,11] and several treatment algorithms have been proposed to guide the clinician in his treatment decision [12,13,14]. Although anti-TNF trough and ADA concentrations correlate with both clinical and endoscopic outcomes, it is still not clear what is the threshold beyond which an optimization would appear futile [15,16].

Commercial assays are readily available for both UST and VDZ and serum concentrations of VDZ and UST have been reported to correlate with patient response [17]. However, little is available to guide clinicians on the optimal timing of dosing and thresholds to aim for with these two biologics compared to anti-TNF biologics, as we still have minimal data from randomized controlled and observational studies [18]. The review summarize the latest available data on TDM, including the pharmacokinetics, clinical and endoscopic outcomes, and its clinical utility for UST, VDZ, and tofacitinib.

## 2. Pharmacokinetics of Ustekinumab

Ustekinumab (Stelara®; Janssen Biotech, Inc., Horsham, PA, USA) has been recently approved to treat both moderate to severe CD [19] and UC [6]. The administration of this drug for induction is intravenous (iv) and weight-based, followed by a subcutaneous (sc) dose injection with a fixed dose for maintenance that could be administered every 4, 8 or 12 weeks depending on the inflammatory burden. As initially demonstrated in psoriasis studies, the bioavailability of UST following sc administration is estimated around 57% [20,21]. The median time to reach the maximum serum concentration after a single sc dose of 45 mg and 90 mg was 13.5 days and 7 days respectively [22].

The elimination half-life of UST varies from 14.9 to 45.6 days. This prolonged half-life reflects the ability of the neonatal Fc receptor to protect antibody proteins from being degraded by lysosomes and thus preventing rapid systemic elimination of IgG [23]. Inter-patient variability related to differences in body weight, serum albumin level, race (Asian versus non-Asian), sex, C-reactive protein (CRP), having failed an anti-TNF treatment and the presence of antibodies against UST [24] appear to be the most important covariates to affect UST’s clearance. Patients with a weight > 100 kg have indeed a median clearance about 55% higher compared to those with a weight ≤ 100 kg. However, contrasting with anti-TNF drugs, the addition of an immunomodulators does not appear to influence significantly UST concentrations as discussed below [25]. There appears to be interdose variation in UST concentrations, thus motivating to do a TDM at the trough of the dose [26].

## 3. Exposure-Response Relationship with Ustekinumab

Exposure-response relationships with anti-TNF medications are often reported as quartile analyses: increasing quartiles of drug concentrations are correlated to better clinical response and remission rates, but also with higher rates of mucosal healing, lower CRP and fecal calprotectin (FCP) levels. These observations seem to apply to UST as well (Table 1).

### 3.1. Exposure-Response Relationship for UST in Crohn’s Disease during Induction

The relationship between UST exposure during the first weeks of treatment (peak concentrations) and outcomes of patients with CD is not perfectly understood. The exposure-response relationship association of UST has been mainly established through the pivotal phase 3 studies called UNITI-1, 2 and IM-UNITI that used a drug tolerant ELISA [5,25,26]. Eight weeks after induction, the median concentration of UST were 2.1 and 6.4 μg/mL respectively for the 130 mg and 6 mg/kg dose groups and serum concentrations of the drug correlated with clinical remission at week 8 in both UNITI-1 and -2 [5,25,28]. UST concentrations from 3.2 to 4 µg/mL were associated with increased rates of clinical remission at eight weeks post induction [26]. In a recent post hoc analysis from the pivotal UNITI studies (UNITI 1, 2), Adedokun et al., reported that serum concentrations of UST were correlated to the dose administrated and the efficacy of the therapy during induction [25].

Interestingly, in a recent prospective study, Hanžel and colleagues investigated the relationship between serum concentrations of UST immediately after IV infusion (as early as 1 h after IV infusion) and at week 2 of treatment with biochemical and endoscopic remission in 41 consecutive CD patients started on UST (6 mg/kg, IV, then 90 mg every eight weeks sc). Endoscopic remission was achieved in forty-six percent (46%) with peak concentrations above 105 mg/mL (upper tercile) compared with only one of 14 patients (7%) with peak concentrations below 88 mg/mL (lower tercile). Therefore, early measurement of serum concentrations of UST (as early as 1 h after IV infusion) could be used to identify patients most likely to achieve endoscopic remission and may be helpful in the future to optimize induction treatment of UST [29].

### 3.2. Exposure-Response Relationship for UST in Crohn’s Disease during Maintenance

Adedokun et al. [25] established from IM-UNITI maintenance study that UST serum concentrations were correlated to dose and were not significantly different compared to the UST induction studies. By the second maintenance dose UST concentration were at a steady state. Interestingly, the median steady-state serum trough UST concentrations measured at week 26 in the group receiving the drug every eight weeks (1.97–2.24 μg/mL) were roughly threefold higher than in the group receiving the drug every 12 weeks (0.61–0.76 μg/mL) with a trend towards higher rate of clinical remission in the Q8 week group. Patients receiving a dose every 12 weeks during maintenance period and experiencing a LOR were also able to recapture response in 55% of cases after an escalation of the administered dose every eight weeks, suggesting a dose-response. Compared to TNF antagonist refractory patients, clinical remission was higher in anti-TNF naïve patients, but UST concentrations in the serum were comparable in both groups [5,28]. Quartile analysis revealed that the concentrations of UST range from 0.92 to 1.2 µg/mL were associated with higher rates of clinical remission both with Q8 and Q12 regimen at week 24 [26].

Overall, a cut-off of approximately 1 μg/mL of UST concentration had an Area under the Curve (AUC) of 0.64 (*p* < 0.003) for predicting clinical remission. Concentrations of UST greater than 1.1 μg/mL were also correlated to higher level of CRP normalization compared to lower concentrations (52% vs. 25%, *p* < 0.0001) after 24 weeks. UST concentrations above 0.5 μg/mL were associated at week 44, in a small subset of 100 patients, with higher endoscopic response rates (40% vs. 8%, *p* < 0.003). The McGill University group reported an observational study of 62 anti-TNF refractory CD patients, induced by a subcutaneous UST regimen, were TDM was measured using a drug tolerant high mobility shift assay (HMSA) (Prometheus, San Diego, CA, USA) [30]. This study suggested that trough serum concentration of UST above 4.5 μg/mL after 26 weeks seem to be associated with biochemical and endoscopic response (67% sensitivity, 70% specificity; AUC, 0.67) [30], but also with a composite outcome encompassing the rate of steroid-free clinical remission and endoscopic response (40.7% below 4.5 μg/mL vs. 75.9% for concentrations above, *p* = 0.008). In contrast, one French prospective study did not report an exposure-response correlation evaluating the association between serum UST trough levels and response to induction and maintenance treatment in 49 anti-TNF refractory CD patients [31].

These discrepancies could be explained by different end points of clinical remission and endoscopic improvement. In addition, measurement of UST and anti-UST antibody concentrations were carried out by different lab assays (ELISA assay and drug-tolerant liquid phase homogeneous mobility shift assay). Verdon et al. compared three different drug testing assays used in Europe, Canada and the USA [32]: Prometheus (drug-tolerant HMSA), Dynacare (ELISA Progenika) and Theradiag (ELISA). They found a good correlation and good agreement between the ELISA tests for serum UST drug concentrations. However, the agreement was poor between the HMSA and each ELISA tests, where the HMSA UST concentrations were approximately twofold higher than ELISA UST concentrations.

Finally, preliminary pharmacological data from the STARDUST (a study of treat to target versus routine care maintenance strategies in Crohn’s disease patients treated with ustekinumab) trial were also recently published. STARDUST is the first treat-to-target (T2T), randomized trial of CD adult patients using endoscopy at week 16 to guide the first decision of UST dose adjustment compared to a standard of care strategy to achieve endoscopic response at week 48 vs. baseline). Median serum UST concentrations at week 0 (1 h postinfusion), week 8 trough, and week 16 trough (112.7 µg/mL, 6.7 µg/mL, and 2.7 µg/mL, respectively) were similar to those from the pivotal CD studies (UNITI-1, UNITI-2, & IM-UNITI) at the same timepoints, irrespective of being biologic naive or not. At week 8 and week 16, the proportion of patients in clinical response and remission were similar across the top three UST concentrations quartiles (Q2–Q4), but lower in the lowest quartile (Q1). Biological markers (CRP and FCP levels) were inversely correlated with UST concentrations at week 8 and week 16. At week 8 and week 16, the proportion of patients with normal biomarkers increased proportionally to UST concentrations. UST concentrations were positively associated with endoscopic response and remission. A trough serum UST concentration of 0.8 to 1.4 μg/mL or greater was associated with clinical remission during maintenance. Serum UST concentrations and the low incidence of antibodies to UST were consistent here with data from pivotal CD randomized controlled trials and previous post hoc analysis. Most patients achieved clinical efficacy outcomes at UST concentrations derived from the approved dose regimen. The inverse association between UST concentrations at week 8 and week 16 and CRP and FCP levels at baseline suggest, as it is the case for anti-TNF, that patients with highest inflammatory burden have faster drug clearance. These patients could benefit from a shorter administration interval.

### 3.3. Exposure-Response Relationship for UST in Ulcerative Colitis

UST was recently approved for use in moderate-to-severe UC. Though there is considerable overlap between UC and CD, these diseases are treated differently and there may be differences in the association between drug levels and clinical response.

Endoscopy is considered a “hard outcome” and potentially more informative and more sensitive for picking up differences in pharmacokinetics. Interestingly, the pivotal CD analyses did not incorporate objective outcomes on endoscopy for all patients but were based mostly on clinical response or remission and biomarkers. In UC study UNIFI, endoscopy was a key secondary outcome and was also incorporated into the primary outcome [6].

Adedokun et al., evaluated the association between serum concentrations of UST and various responses in patients with moderate-to-severe UC enrolled in the UNIFI trial [33]. They found that serum concentrations of UST are proportional to dose consistently among patients with CD and UC and correlate with clinical and histologic efficacy and markers of inflammation. In patients with UC, serum concentrations of UST were dose-proportional and were at a steady state concentration by the second maintenance dose. Compared to every 12-week dosing, the median trough UST concentration was threefold higher with every eight weeks. Serum concentrations were correlated with clinical, biochemical and histologic remission. A week 8 post induction concentration on 3.7 μg/mL was associated with response, and a maintenance concentration of 1.3 μg/mL was correlated with increased rates of clinical remission. Higher serum concentrations of UST were not associated with any adverse events.

More recently, the UNIFI long-term extension study results were published [34]. The efficacy and safety of UST was evaluated among patients who received UST sc in the long-term extension of UNIFI (two years). Treatment with UST 90 mg sc q12w or q8w over the two years results in sustained serum UST concentration and consistent with serum UST levels observed in the UNIFI maintenance study.

In conclusion, except for one conflicting study, there is a clear exposure-response association with UST in the current literature. Commercial assays are available for this drug in clinical practice and the exact threshold below which dose optimization may be useful still need to be determined. On the basis of data previously presented, experts agree to target UST concentration of at least 3–7 μg/mL at week 8 and 1–3 μg/mL during maintenance for both CD and UC [35] (Table 2).

## 4. Dose Escalation with UST

UST dose optimization by decreasing the interval of administration or IV reinduction are both strategies that can be employed to establish remission and response in patients with IBD with partial response or LOR to UST maintenance therapy. Data on dose escalation from UST 90 mg q8w to UST 90 mg q4w demonstrate the efficacy of dose escalation in patients with partial LOR to UST therapy.

In a small cohort study of 38 patients with anti-TNF refractory severe CD, escalating dose of UST from every eight weeks to every four weeks was reported in approximately 50% of the patients and clinical response was attained in 61.1% [36]. One hundred and sixteen consecutive CD patients with CD were included in another study across 42 tertiary IBD center from Spain. The authors described that 84% of the patient reached clinical response after induction with UST, dose escalation was necessary for 10% of the cohort and was successful in 73% of them. Dose escalation has now demonstrated its benefits to improve clinical response and a TDM approach to guide optimization with UST has be proposed.

Another Canadian group reported a prospective study were dose escalation from q8w to q4w was performed in 22 patients. Seventy-six percent showed a response or remission at a median follow-up visit after 3.8 months (45% remission, 31% response) [37]. In addition, Fumery et al. presented data showing a response in two thirds of patients and clinical remission in 41% of patients after dose escalation. Dose optimization was performed in 26% of patients due to LOR and in 74% of patients due to inadequate response [38]. These results were also confirmed by a cohort study from the French GETAID group where, among 66 active CD optimized to 90 mg every four weeks, two-thirds recaptured response following optimization [39].

More recently a multicenter retrospective cohort study was performed across five sites in Canada and Switzerland to evaluate the efficacy of an IV dose re-induction of UST (approximately 6 mg/kg) in 65 patients for either partial response or secondary LOR to UST, based on clinical, biochemical or endoscopic criteria. 88.3% were already optimized on q4w priori reinduction [40]. Clinical outcomes were analyzed at a median of 14 weeks (IQR: 12–19) post reinduction. Clinical remission off corticosteroids with biochemical and endoscopic response or remission was achieved in 31.0% of patients (*n* = 18). Pre-reinduction UST concentrations were ≥1 μg/mL in 88.6% (mean 3.2 ± 2.0 μg/mL) without any serious adverse events reported following UST reinduction.

A prospective, interventional study is required to address whether trough serum UST concentrations optimization via TDM improves efficacy outcomes. Several studies are underway looking at different optimization protocols including. The results of these studies are needed before firm recommendations can be given on how and when to optimize treatment with UST.

## 5. Combination Therapy and Immunogenicity

Combination therapy with a thiopurine or methotrexate and anti-TNF is well-established in moderate-to-severe UC and CD to decrease immunogenicity. However, whether patients with IBD treated with non-anti-TNF biologics should receive concomitant thiopurines or methotrexate remains controversial.

To date, data suggests that UST efficacy in CD and UC did not differ in patients who were on or not on concomitant immunosuppressive treatment. Similarly, the addition of an immunomodulator with UST have no significant benefit on pharmacokinetics, as the concentrations are similar between groups of patients receiving and not receiving concomitant treatment with UST [26,41,42]. A meta-analysis of 31 studies (randomized controlled trial, *n* = 6; cohort studies, *n* = 25) of IBD patients treated with UST or VDZ was recently published. In this study, combination therapy was no more effective than monotherapy as induction or maintenance therapy [43].

Although it is possible that UST can be used effectively as monotherapy those studies have limitations (e.g., lack of baseline data, immunomodulator doses not reported, and inability to control for unmeasured confounders). To date, pharmacokinetic data have been based on subgroup analyses and the studies from which this meta-analysis was drawn were not designed to assess this issue. Therefore, validation in a second larger dataset or a formal RCT is required, as possible confounders may have influenced the choice of monotherapy or combination therapy.

The lack of benefit from combination therapy with UST may be a reflection of lower immunogenicity rates; only 0.7% of the 427 patients from the CERTIFI trial developed antibodies against UST. However, follow-up was only 36 weeks and a drug sensitive ELISA, which is unable to detect antibodies in the presence of drug, was used [44]. In the IM-UNITI study, the rate of UST antibody formation using a drug-tolerant assay was only 2.3% [5]. In the UNIFI long-term extension study, the rate of antibody formation was quite low at 5.5% (22/400) and were often transient in nature. Most patients (18/22) had very low titers (below 1:800) and only four had neutralizing antibodies. There was no association between patients in clinical remission or injection site reactions and the presence of antibodies to UST [34]. These low rates of UST antibodies contrast with the higher rate of antibodies (between 14–73% depending on the assay methodologies used and the influence of patient/drug-related factors) observed with the anti-TNF drugs [45].

In conclusion, UST concentrations of 3–7 μg/mL at week 8 and 1–3 μg/mL during maintenance have been associated with improved outcomes and dose optimization generally improves clinical outcomes in those with partial response or LOR (Table 2). Therefore, UST TDM may be of clinical use both in the reactive and proactive setting, but clear cut-offs as to when dose escalation would be futile are presently lacking and further studies need to be completed to optimize the use of TDM with UST.

## 6. Vedolizumab

Vedolizumab (VDZ) (Entyvio TM^®^, Takeda Pharmaceuticals U.S.A.) is a humanized IgG1 monoclonal antibody which targets solely the α4β7 integrin. The α4β7 integrin is responsible for the modulation of lymphocyte trafficking in the gut and its selective blocking prevents a systemic immunosuppression. The pivotal GEMINI studies confirmed the efficacy of VDZ in patients with UC and CD in both anti-TNF naïve and exposed patients.

VDZ is administered by infusion at a nonweight based dose of 300 mg IV for the induction phase (at zero, two and six weeks), and during the maintenance phase every eight weeks. The recent results from VISIBLE 1 confirmed the efficacy and safety of VDZ with a new sc formulation in UC and the results from the VISIBLE 2 (NCT02611817; EudraCT 2015-000481-58) study of VDZ sc in CD were recently presented [46]. Maintenance therapy with the VDZ sc formulation confers efficacy and safety similar to the therapeutic profile of the IV formulation in patients with UC and CD who respond to IV induction [47].

It terms of pharmacokinetics, the median serum VDZ was higher in those patients receiving sc dosing (39.8 μg/mL, 90% CI, 20.8–75.4 μg/mL) versus IV dosing at 32.2 μg/mL (90% CI, 16.5–60.7 μg/mL). Quartile analysis of drug concentrations during maintenance therapy demonstrated an exposure response with clinical remission increasing from 50% in the first quartile, compared to 83% in the fourth quartile. The rate of anti-VDZ antibodies was low at 6%, in patients receiving either SC or IV VDZ, and the rate of neutralizing antibodies was 3% in both groups. There was lower exposure and reduced treatment efficacy in those patients that developed anti-VDZ antibodies.

## 7. Pharmacokinetics of Vedolizumab

Multiple determinants contribute to VDZ pharmacologic profile. The α_4_β_7_ integrin is expressed on a subcategory of leukocytes, thus involving that the drug does not fix to the majority of memory CD4^+^ T lymphocytes, neutrophils and monocytes [48] and the maximum concentration of binding concern only around 25% of memory CD4^+^ T lymphocytes in the peripheral blood including gut-homing interleukin 17 T-helper lymphocyte. In addition, the drug inhibits lymphocyte movement into the gastrointestinal tract by binding to the α4β7 integrin only and not α4β1 and αEβ7 integrins [48]. Finally, there is gastrointestinal-specific tropism of the α4β7 integrin function. These pharmacologic properties of the drug limit to the gastrointestinal (GI) system the exposure of VDZ in the body. This good risk-benefit profile is also particularly interesting for more fragile IBD patients [17].

VDZ has a slow linear elimination until approximately 10 μg/mL but the elimination process is faster and nonlinear at lower concentrations [49,50]. The half-life and clearance of VDZ is not modified at doses >2.0 μg/mL and the increase in exposure is proportional to drug’s dose [49,50]. Elimination is possible through cellular uptake followed by proteolytic degradation and the clearance is receptor-mediated. Comparable to UST, VDZ has a prolonged half-life of around 25.5 days during this linear elimination because it binds to the neonatal Fc receptor protecting the antibody from proteolysis. Clearance appears comparable for both CD and UC [49,50,51] but increased for people with severe obesity (>120 kg) and low levels of albumin (<3.2 g/dL) [51,52]. As observed with anti-TNF-a drugs, increased inflammatory load and development of neutralizing antibodies augmented VDZ clearance [51]. Concomitant immunosuppressive drug (methotrexate and thiopurines) do not seem to have a clinical relevant effects on VDZ clearance [49,50,51,52].

The saturation of the α4β7 receptor happens when serum VDZ drug levels is low, and measuring it alone is therefore insufficient to predict clinical outcomes. Interestingly studies have shown that dosing of VDZ at every eight weeks saturates more than 95% of the α4β7 receptors on peripheral lymphocytes [53]. This indicates that increasing concentrations of VDZ when the target receptor is already saturated would not be associated with better efficacy. Recent in vitro and in vivo works support the concept that there is a dose-dependent differential binding of VDZ to different T-cell subpopulations suggesting that an optimal ‘window’ of exposure exists [54].

Interestingly, the higher efficacy of increased concentrations of VDZ was demonstrated in numerous studies, indicating that target receptor saturation is not the sole reason linked to improvement in clinical outcomes [51]. The pharmacokinetic data and the long dosing interval of the drug explain why VDZ TDM should be performed at dosing trough, like IFX and UST.

## 8. Exposure-Response Relationship for VDZ in Ulcerative Colitis and Crohn’s Disease

The utility of VDZ TDM in patients with UC or CD is unclear but pharmacokinetic data suggest that VDZ concentrations correlate with clinical outcomes, although the correlation seems less strong for VDZ compared with anti-TNF agents (Table 1). Current available data are quite heterogenous and the clinical utility of VDZ drug monitoring is still under investigation [55].

### 8.1. Exposure-Response Relationship for VDZ during Induction

The association between VDZ exposure and clinical efficacy in IBD was initially reported in the GEMINI studies [2,3]. The relationship between clinical outcomes in UC and serum concentrations of VDZ measured by an ELISA test was described in the GEMINI 1 trial [2]. In this study serum concentrations measured at week 6 were comparable between the lowest quartile of concentrations (<17 μg/mL) and the placebo group, with only a 6% remission rate. Conversely, patients in the highest quartile (>35.7 μg/mL) had significantly better outcomes compared to subjects in the lowest quartile with 37% of remission rate [2] and 62.9% of mucosal healing [56]. The GEMINI 2 and GEMINI 3 trials also reported an association between trough concentrations at week 6 and clinical outcomes, although this association was less strong with only 22% of remission rate in the highest quartile (>33.7 μg/mL) compared to 6% in the placebo group and the lowest quartile in GEMINI 2 [3]. The median serum VDZ concentrations at week 14 obtained by a drug tolerant high mobility shift assay were superior in a cohort of 35 patients with IBD in the responders group compared to the nonresponders group (12.3 vs. 7.1 μg/mL, *p* = 0.02) but also higher among steroid-free versus steroid-dependent IBD patients (12.5 vs. 7.8 μg/mL, *p* = 0.03) [57].

A post hoc analysis of the GEMINI 1 trial reported also that higher trough concentrations of VDZ (>38.30 µg/mL) at week 6 were associated with clinical remission at week 14 [58]. Higher median trough concentrations of VDZ at weeks 2 (>35.60 µg/mL) and 4 (>59.40 µg/mL) were also associated with higher clinical remission rates at week 14, compared to patients not in clinical remission [58]. In a small group of 29 patients, trough level higher than 40 μg/mL at week 6 on an ELISA assay (Theradiag, France) was associated with clinical remission beyond 28 weeks, with a sensitivity of 100% a specificity of 70% and AUC of 0.84 [59].

In another study, VDZ concentrations obtained by a drug sensitive ELISA (Theradiag, France) was prospectively measured in 47 patients with IBD. Here again, higher trough concentrations of VDZ (41.7 μg/mL vs. 21 μg/mL *p* = 0.07) were associated with clinical remission at week 6 [60]. The authors reported that a cut-off level of 37 µg/mL was associated with remission at week 6 with a sensitivity of 100% and specificity of 70%. The same group recently published data indicating that a trough level of VDZ below 19 μg/mL at week 6 predicted the need for optimization (AUC 0.72) to Q4 week dosing from week 10. Optimization to Q4 weeks at week 10 resulted in the entire group of patients with low trough concentrations being in clinical remission at week 14 [61].

Dressen et al., observed a correlation between VDZ exposure and response and identified patient factors that affect exposure and response in 179 patients with CD or UC. Serum concentrations of VDZ were measured before all infusions up to week 30 and effectiveness endpoints (clinical, biological and endoscopic) were evaluated at week 14 for UC patients and week 22 for CD patients.

Trough concentrations of VDZ above 30.0 μg/mL at week 2, 24.0 μg/mL at week 6, and 14.0 μg/mL during treatment maintenance were associated with a higher rate of clinical efficacy (*p* < 0.05). Increased body mass and more severe biochemical inflammation (increased CRP/decreased albumin) at week 0 were associated with lower trough concentrations of VDZ and a lower likelihood of achieving mucosal healing (*p* < 0.05) [62].

Recently, an observational study in anti-TNF naïve and exposed patients suggested a clear exposure-endoscopic response relationship exists for VDZ. VDZ trough concentrations were measured at week 6, week 14 and during maintenance in 336 patients, 20% being anti-TNF naïve. A better endoscopic outcome was associated with significantly higher drug exposure in both CD and UC. However, not all patients benefit from treatment intensification [63].

The retrospective cohort study called ERELATE included 695 IBD patients (391 CD and 304 UC) from nine tertiary centers in six countries [64]. This work was in line with the findings of GEMINI trials where a significant relationship between VDZ trough concentration and clinical outcomes was observed. The exposure-response relationship was more marked in UC than CD. Predicted concentrations of VDZ between 30.8 and 33.8 µg/mL at week 6 correlated with clinical deep remission at week 52. Therefore, a sufficient concentration of VDZ during the induction phase may be an important predictor of short- and long-term outcomes with this treatment.

Based on quartile analysis, there appears to be an exposure-response relationship of VDZ drug concentration during induction, and a drug level above 33–37 μg/mL at week 6 is now recommended by experts’ consensus [52].

### 8.2. Exposure-Response Relationship for VDZ during Maintenance

In both the GEMINI 1 and 2 trials, patients in the highest VDZ concentration quartile had 20% increase in clinical remission at week 54 compared to those in the lowest quartile (<6 μg/mL, every eight weeks at week 48 in GEMINI 1 and <7.5 μg/mL, every eight weeks at week 48 in GEMINI 2) [12,44]. Interestingly, this exposure-response relationship was unclear in both trials among patients receiving VDZ every four weeks [12,44].

In a prospective cross-sectional study, 56 IBD patients received VDZ as a maintenance treatment and VDZ concentrations were measured by a drug-tolerant assay. Once again, patients in deep remission, defined by normal CRP and Simple Endoscopic Score for Crohn Disease (SES-CD) score, and in steroid-free remission, had significantly higher levels compared to patients who did not achieve these outcomes (12.9 vs. 9.4 μg/mL (*p* = 0.008), and 15 vs. 9.5 (*p* = 0.02)). Patients in the first interquartile threshold of VDZ levels (≥5.1 μg/mL) had higher chance to reach deep remission (OR: 6.6 (95% CI: 1.55–45.8) *p* = 0.009) and the cutoff of 5.1 μg/mL was the best predictor of deep remission (ρ: 0.713, *p* = 0.03) [65].

Similar results on the dose-exposure relationship are also observed in the IBD pediatric population during maintenance treatment and a study including 113 patients on VDZ reported that levels below the median (11 μg/mL) were significantly less prone to lead to clinical remission compared to higher levels (37.9% versus 61.8%, *p* = 0.01) [66].

A large real-world multicenter cohort including 258 IBD patients in remission evaluated VDZ concentrations during maintenance measured by homogeneous mobility shift assay (HMSA). Remission rates were significantly higher among patients with superior trough VDZ concentrations (12.7 µg/mL vs. 10.1 µg/mL, *p* = 0.002) and among patients in endoscopic and deep remission (14.2 µg/mL vs. 8.5 µg/mL, *p* = 0.003 and 14.8 µg/mL vs. 10.1 µg/mL, *p* = 0.01, respectively). Patients with trough VDZ concentrations superior to 11.5 µg/mL were nearly 2.4 times more likely to be in corticosteroid-free clinical and biochemical remission after controlling for potential confounders [67]. The ERELATE real-word study has shown that predicted VDZ concentrations of 16.6 at week 14 were associated with clinical remission and 14.4 µg/mL at week 52 with deep remission [64].

In summary, cumulative evidence from existing literature suggests that an exposure–efficacy relationship may exist for VDZ in IBD. VDZ assays are currently commercialized and can be used in clinical practice. It is still important that clear thresholds for clinical use be determined. Based on the latest literature experts recommend targeting VDZ concentrations of 33–37 μg/mL at week 6, 15–20 μg/mL at week 14 (end of induction period) and 10–15 μg/mL during maintenance to improved clinical outcomes.

## 9. Dose Escalation with VDZ

Similar to UST, there is limited data demonstrating that dose optimization, in patients with low trough VDZ levels experiencing secondary LOR, improves outcomes.

Dose escalation by increasing dosing frequency from eight to every four weeks (GEMINI-long-term study) in patients on maintenance therapy with secondary LOR, who had withdrawn early from GEMINI-2 trial has been reported to increase rates of clinical remission (32% vs. 4% remission before dose increase) [68].

Peyrin-Biroulet et al. [69] observed in a meta-analysis that the pooled incidences rates of LOR to VDZ were 47.9 per 100 person-years of follow-up evaluation among CD patients and 39.9 per 100 person-years of follow-up evaluation among UC patients. Secondary nonresponders with dose optimization regain response to VDZ in 53.8% of cases.

Another Belgian study of 62 IBD patients with secondary LOR to VDZ assessed the impact of dose optimization on serum trough levels and subsequent recapture of response. The median VDZ trough concentration increased from 8.8 μg/mL (interquartile range (IQR), 5.1–13.5 μg/mL) (T0) to 19 μg/mL (IQR, 11.9–22.9 μg/mL) (T1) and 23.1 μg/mL (IQR, 15.5–28.4 μg/mL) (T2) (all *p* < 0.0001) after dose optimization [70]. Biological and clinical response was also observed in 44% and 59% of patients, respectively, but did not correlate with an increase in trough level.

## 10. Combination Therapies and Immunogenicity

There are no randomized controlled trials comparing the efficacy of VDZ monotherapy with combination therapy of VDZ and an immunomodulator. Associations between concomitant use of immunomodulators and decreased immunogenicity of VDZ have been described, but without enhancing the therapeutic effect of therapy. The combination of VDZ with an immunomodulator does not seem to affect the clearance of the drug or the efficacy [2,3]. Further comparative effectiveness studies of patients with IBD, naïve to VDZ as well as immunomodulators are required.

In terms of immunogenicity, given that a drug-sensitive ELISA assay was used, the antibody formation rate could not be accurately ascertained in the pivotal VDZ clinical trials [50,71]. Immunogenicity was uncommon but can develop, as 12% of patients that discontinued VDZ (placebo maintenance arm) formed antibodies [72]. The rates of antibody formation have been also quite low (<5%) in real world cohorts where drug-sensitive or drug-tolerant assays were used [51,59,66].

In summary, VDZ concentrations of 33–37 μg/mL at week 6, 15–20 μg/mL and week 14 and 10–15 μg/mL during maintenance have been associated with improved outcomes (Table 2). Although there is less data than with UST, dose optimization may improve clinical outcomes, in those with partial response or LOR. VDZ TDM may be of use in the reactive and proactive setting, but clear cut-offs of when dose escalation would be futile are presently lacking and further studies need to be completed to optimize the use of TDM with VDZ.

## 11. Tofacitinib

Tofacitinib is an oral small-molecule pan-JAK inhibitor recently commercialized. This drug has proven to be effective in both induction and maintenance periods in moderately-to-severe active UC patients. In the pivotal OCTAVE trials, a higher percentage of patients randomized to 10 mg twice daily reached the primary endpoint of remission during maintenance period (Week 52), compared to patients randomized to 5 mg twice daily group [73]. This is similar to an earlier phase 2 trial in patients with UC where a dose-dependent effect was detected during induction phase [74]. These results suggest that some patients could benefit from superior doses of tofacitinib. In the psoriasis literature, pharmacokinetic data of tofacitinib reported that heavier patients who had been exposed to biologic treatments required higher dose of tofacitinib to achieve favourable outcomes [75].

In moderately-to-severely active UC patients, a population pharmacokinetic analysis revealed that plasma concentrations of tofacitinib increased proportionately with dose. There were no differences in tofacitinib concentrations at baseline versus at the end of induction at week 8 [76]. In terms of clearance, the interpatient variability was 31.4%, which is what has been demonstrated for other biologic treatments. There was no significant correlation with any clinical or biochemical measure at baseline. Although these results are reassuring, further studies need to be completed to explain the interindividual variability seen in clearance in UC patients. In another study, the baseline Mayo score was associated with week 8 outcomes but tofacitinib concentrations were not predictive of efficacy, beyond what was provided with the dose of tofacitinib [76].

## 12. Conclusions

Primary nonresponse, partial response and LOR are affected by variety of patient and disease-related elements, influencing the pharmacokinetics and dynamics of biologics. Reactive TDM has become the standard to care for anti-TNF medications and proactive TDM is increasingly being performed, even with conflicting data regarding its utility in clinical practice. The impact of measuring drug concentrations and ADA levels for newer treatments such as VDZ, UST and tofacitinib is less clear than for anti-TNF’s. Although an exposure-response relationship for these novel agents has been now established in both CD and UC, the value of TDM for optimizing treatment with these agents is still to be determined, particularly given differences regarding the mode of action, drug pharmacokinetics and immunogenicity compared to anti-TNF drugs. Dose optimization is an efficient in regaining clinical response and remission in UST, VDZ and tofacitinib. Given clinicians thirst for any information that may help with optimizing clinical treatment, TDM testing for UST and VDZ will no doubt increase, and this review aimed to educate clinicians on current available TDM data. New clinical trials will need to confirm that reactive and proactive dose optimization, based on TDM, improve overall outcomes and is also a cost-effective strategy.

## Figures and Tables

**Table 1:** Serum biologic drug concentration thresholds can vary based on inflammatory bowel disease (IBD) phenotype and the desired therapeutic outcome to target. Adapted from [27].

Drug Type	IBD Type	Time Point (Week)	Threshold (μg/mL)	Therapeutic Outcome (Time Point)	TDM Assay	References
VDZ	UC	2	>28.9	Clinical response (week 14)	ELISA	Dreesen et al., 2018
VDZ	UC	2	>23.7	Mucosal healing (week 14)	ELISA	Dreesen et al., 2018
VDZ	CD	6	>13.8	Endoscopic remission (6 months)	ELISA	Verstockt et al., 2020
VDZ	CD	6	>19.9	Biological remission (6 months)	ELISA	Verstockt et al., 2020
VDZ	UC	6	>20.9	Endoscopic improvement (week 14)	ELISA	Verstockt et al., 2020
VDZ	UC	6	>23.9	Clinical remission (week 14)	ELISA	Verstockt et al., 2020
VDZ	CD	14	>30.1	Endoscopic improvement (6 months)	ELISA	Verstockt et al., 2020
VDZ	CD	14	>21.2	Clinical remission (6 months)	ELISA	Verstockt et al., 2020
VDZ	CD	14	>25.2	Biological remission (6 months)	ELISA	Verstockt et al., 2020
VDZ	UC	14	>10.1	Endoscopic improvement (week 14)	ELISA	Verstockt et al., 2020
VDZ	UC	14	>10.1	Clinical remission (week 14)	ELISA	Verstockt et al., 2020
VDZ	UC	14	>6.8	Biological remission (week 14)	ELISA	Verstockt et al., 2020
VDZ	UC	14	>12.6	Clinical response (week 14)	ELISA	Dreesen et al., 2018
VDZ	UC	14	>17	Mucosal healing (week 14)	ELISA	Dreesen et al., 2018
VDZ	CD	22	>13.6	Mucosal healing (week 22)	ELISA	Dreesen et al., 2018
VDZ	CD	22	>12	Biological remission (week 22)	ELISA	Dreesen et al., 2018
VDZ	CD/UC	Maintenance	>11.5	CS-free clinical & biochemical remission	HMSA	Ungaro et al., 2019
VDZ	CD/UC	Maintenance	>10.7	CS-free endoscopic remission	HMSA	Ungaro et al., 2019
VDZ	CD/UC	Maintenance	>14.8	CS-free deep remission	HMSA	Ungaro et al., 2019
VDZ	CD	Maintenance	>6.8	CS-free clinical & biochemical remission	HMSA	Ungaro et al., 2019
VDZ	UC	Maintenance	>10.1	CS-free clinical & biochemical remission	HMSA	Ungaro et al., 2019
VDZ	CD	Maintenance	>12.1	Biological remission after 6 months	ELISA	Verstockt et al., 2020
VDZ	CD	Maintenance	>10.1	Endoscopic remission after 6 months	ELISA	Verstockt et al., 2020
UST	CD	4	>15	FC < 100 mg/g (week 16)	ELISA	Hanzel et al., 2020
UST	CD	4	>23.7	Endoscopic remission (week 24)	ELISA	Hanzel et al., 2020
UST	CD	8	>4.2	50% decrease in FC (week 8)	ELISA	Verstockt et al., 2019
UST	CD	8	>7.2	Biological remission (week 8)	ELISA	Verstockt et al., 2019
UST	CD	8	>4.4	FC < 100 mg/g (week 16)	ELISA	Hanzel et al., 2020
UST	CD	8	>6.9	FC < 100 mg/g (week 24)	ELISA	Hanzel et al., 2020
UST	CD	8	11.1	Endoscopic remission (week 24)	ELISA	Hanzel et al., 2020
UST	UC	8	>3.7	Clinical response (week 8)	ELISA	Adedokun et al., 2019
UST	UC	8	>3.5	Endoscopic improvement (week 8)	ELISA	Adedokun et al., 2019
UST	UC	8	>3.7	Histologic improvement (week 8)	ELISA	Adedokun et al., 2019
UST	UC	Maintenance	≥1.3	Clinical remission (week 44)	ELISA	Adedokun et al., 2019
UST	UC	Maintenance	≥1.1	Endoscopic improvement (week 44)	ELISA	Adedokun et al., 2019
UST	UC	Maintenance	≥1.3	Histologic improvement (week 44)	ELISA	Adedokun et al., 2019

**Table 2:** Simplified summary of trough concentrations to target associated with outcome’s improvement.

Drug	Timing	Trough Drug Concentrations (μg/mL) Associated with Outcome’s Improvement	Outcome Measure
UST	Induction (Week 8)	>3–7	Clinical
UST	Maintenance	>1–3	Clinical/Endoscopic
VDZ	Induction (Week 6)	>33–37	Clinical/Endoscopic
VDZ	End of induction (Week 14)	>15–20	
VDZ	Maintenance	>10–15	Clinical/Endoscopic
